# Adherence to the Mediterranean Diet and Obesity-Linked Cancer Risk in EPIC

**DOI:** 10.1001/jamanetworkopen.2024.61031

**Published:** 2025-02-25

**Authors:** Inmaculada Aguilera-Buenosvinos, Fernanda Morales Berstein, Esther M. González-Gil, Laure Dossus, Marc J. Gunter, Carine Biessy, Giovanna Masala, Maria Santucci De Magistris, Nasser Laouali, Sanam Shah, Chloé Marques, Alicia K. Heath, Konstantinos K. Tsilidis, Amanda J. Cross, Pietro Ferrari, Carlota Castro-Espin, Charlotte Debras, Rosario Tumino, Anne Tjønneland, Jytte Halkjær, Isabel Drake, Ulrika Ericson, Marcela Guevara, Miguel Rodríguez-Barranco, Guri Skeie, Tonje Braaten, Inger Torhild Gram, Christina C. Dahm, Claudia Agnoli, Matthias B. Schulze, José María Huerta, Miguel Ángel Martínez-González, Inge Huybrechts, Estefania Toledo Atucha

**Affiliations:** 1 Department of Preventive Medicine and Public Health, University of Navarra, Pamplona, Spain; 2 Navarra Institute for Health Research, Pamplona, Spain; 3 Medical Research Council Integrative Epidemiology Unit, University of Bristol, Bristol, United Kingdom; 4 Population Health Sciences, Bristol Medical School, University of Bristol, Bristol, United Kingdom; 5 Nutrition and Metabolism Branch, International Agency for Research on Cancer, World Health Organization, Lyon, France; 6 Department of Epidemiology and Biostatistics, School of Public Health, Imperial College London, London, United Kingdom; 7 Institute for Cancer Research, Prevention and Clinical Network, Florence, Italy; 8 Azienda Ospedealiera Universitaria Federico II, Naples, Italy; 9 Université Paris-Saclay, University of Versailles Saint-Quentin-en-Yvelines, Institut National de la Santé et de la Recherche Médicale “Exposome, Heredity, Cancer and Health” Team, Centre for Research in Epidemiology and Population Health U1018, Gustave Roussy, Villejuif, France; 10 Institut National de la Santé et de la Recherche Médicale “Exposome, Heredity, Cancer and Health” Team, Centre for Research in Epidemiology and Population Health U1018, Gustave Roussy, Villejuif, France; 11 Department of Hygiene and Epidemiology, University of Ioannina School of Medicine, Ioannina, Greece; 12 Cancer Screening and Prevention Research Group, Department of Surgery and Cancer, Imperial College London, London, United Kingdom; 13 Unit of Nutrition and Cancer, Catalan Institute of Oncology, L’Hospitalet de Llobregat, Barcelona, Spain; 14 Nutrition and Cancer Group, Epidemiology, Public Health, Cancer Prevention and Palliative Care Program, Bellvitge Biomedical Research Institute, L’Hospitalet de Llobregat, Barcelona, Spain; 15 Hyblean Association for Epidemiological Research AIRE-ONLUS, Ragusa, Italy; 16 Danish Cancer Institute, Copenhagen, Denmark; 17 Department of Public Health, University of Copenhagen, Copenhagen, Denmark; 18 Department of Clinical Sciences in Malmö, Lund University, Malmö, Sweden; 19 Skåne University Hospital, Malmö, Sweden; 20 Diabetes and Cardiovascular Disease, Genetic Epidemiology, Department of Clinical Sciences in Malmö, Lund University, Malmö, Sweden; 21 Instituto de Salud Pública y Laboral de Navarra, Pamplona, Spain; 22 Centro de Investigación Biomédica en Red de Epidemiología y Salud Pública, Madrid, Spain; 23 Escuela Andaluza de Salud Pública, Granada, Spain; 24 Instituto de Investigación Biosanitaria, Granada, Spain; 25 Department of Community Medicine, The Arctic University of Norway, Tromsø, Norway; 26 Department of Public Health, Aarhus University, Aarhus, Denmark; 27 Epidemiology and Prevention Unit, Department of Research, Fondazione Istituto di Ricovero e Cura a Carattere Scientifico Istituto Nazionale dei Tumori, Milan, Italy; 28 Department of Molecular Epidemiology, German Institute of Human Nutrition, Nuthetal, Germany; 29 Department of Epidemiology, Murcia Regional Health Council, Biomedical Research Institute of Murcia, Murcia, Spain; 30 Department of Nutrition, Harvard T.H. Chan School of Public Health, Harvard University, Boston, Massachusetts; 31 Centro de Investigación Biomédica en Red de Fisiopatología de la Obesidad y Nutrición, Madrid, Spain

## Abstract

**Importance:**

Adherence to the Mediterranean Diet (MedDiet) has been associated with a lower incidence of cancer and reduced weight gain. These associations suggest a potential role for the MedDiet in lowering the risk of obesity-related cancers (ORCs). Obesity is a known risk factor for various cancers and shows an inverse association with MedDiet adherence.

**Objective:**

To examine the association between adherence to the MedDiet and the risk of ORCs, considering the possible mediating role of adiposity.

**Design, Setting, and Participants:**

This prospective cohort study analyzed data from the European Prospective Investigation Into Cancer and Nutrition (EPIC) study, which enrolled participants aged 35 to 70 years from 1992 to 2000 across 23 centers in 10 countries. The data analysis was conducted from March 1 to May 31, 2023.

**Exposures:**

Dietary intake before baseline was evaluated using country-specific, validated questionnaires administered at recruitment. Adherence to the MedDiet was scored on a 9-point scale and categorized as low (0-3 points), medium (4-6 points), or high (7-9 points).

**Main Outcomes and Measures:**

The primary outcome was the incidence of ORCs, classified according to the 2015 International Agency for Research on Cancer criteria. Multivariable Cox proportional hazards regression models were used to assess the association between MedDiet adherence and ORC incidence. Mediation analyses were conducted to evaluate the role of waist to hip ratio and body mass index in this association.

**Results:**

A total of 450 111 participants were included in the study (mean [SD] age, 51.1 [9.8] years; 70.8% women) and followed up during a median (IQR) time of 14.9 (4.1) years. Among participants, 4.9% experienced an ORC (rates, 0.053, 0.049, and 0.043 per person-year in the low, medium, and high MedDiet adherence groups, respectively). Participants with high adherence to the MedDiet (7-9 points) had a lower risk of ORC compared with those with low adherence (0-3 points) (hazard ratio [HR], 0.94; 95% CI, 0.90-0.98). A similar inverse association was observed for participants with medium adherence (4-6 points vs 0-3 points). However, mediation analyses did not show associations of waist to hip ratio or body mass index between MedDiet adherence and ORC risk.

**Conclusions and Relevance:**

These findings indicate that higher adherence to the MedDiet is associated with a modest reduction in the risk of ORCs, independent of adiposity measures. Further research is needed to clarify the mechanisms by which the MedDiet may contribute to cancer prevention.

## Key Points

**Question:**

What is the association between adherence to the Mediterranean diet and the risk of obesity-related cancers, and is this association mediated by adiposity?

**Findings:**

In this cohort study of 450 111 participants from European countries, high adherence to the Mediterranean diet was associated with a 6% lower risk of obesity-related cancers compared with low adherence. Furthermore, this inverse association was not mediated by body mass index and waist to hip ratio.

**Meaning:**

These findings suggest that higher adherence to the Mediterranean diet may be linked to a slightly reduced risk of obesity-related cancers.

## Teaser

This cohort study examines the association between Mediterranean diet adherence and risk of obesity-related cancer among European Prospective Investigation Into Cancer and Nutrition (EPIC) participants and the mediating role of adiposity.

## Introduction

The prevalence of excess body weight and the associated cancer burden have increased globally over recent decades. Between 1975 and 2016, the prevalence of excess weight in adults (aged ≥20 years) rose from approximately 21% in men and 24% in women to nearly 40% in both sexes.^1,2^ Currently, 39% of the global population is obese or overweight, despite extensive efforts to curb this epidemic.^3^ According to the International Agency for Research on Cancer, excess body weight is convincingly linked to a heightened cancer risk at 13 anatomic sites, including cancers of the endometrium, esophagus, kidney, pancreas, liver, and breast, among others.^4^

Evidence from epidemiologic studies and clinical trials supports the traditional Mediterranean diet (MedDiet) for its positive influence on health, including associations with weight loss and reduced abdominal adiposity.^5,6,7,8,9^ For instance, the European Prospective Investigation Into Cancer and Nutrition (EPIC)-Spain cohort study found an inverse association between high MedDiet adherence and obesity risk among individuals with overweight.^10^ Romaguera et al^10^ also observed that adherence to the MedDiet was associated with a smaller waist circumference and could prevent weight gain in European populations. Additionally, a recent study by Castro-Espin et al^11^ found that adherence to the MedDiet is associated with improved survival after a breast cancer diagnosis in women across 9 European countries, further underscoring the MedDiet’s protective role in cancer prognosis.

The benefits of MedDiet adherence may extend beyond reducing abdominal fat. In the EPIC study, Couto et al^12^ found that higher MedDiet adherence was linked to a 4% reduction in overall cancer risk per 2-point increase in the MedDiet score, with the strongest associations for colorectal, gastric, and breast cancers, particularly when alcohol was excluded from the score. A meta-analysis also found that higher MedDiet adherence was associated with lower cancer mortality and specific cancer incidence.^13^ Similarly, researchers within Italian EPIC centers observed a protective association of the MedDiet with colorectal cancer risk, though abdominal adiposity did not mediate this association.^14^ Limited studies have examined the role of adiposity as a mediator in the MedDiet’s associated outcomes in obesity-related cancers (ORCs). Mechanisms linking obesity and cancer are complex and include factors such as adipokines, growth factors, and insulin resistance, as well as emerging factors such as hypoxia, genetic susceptibility, stromal cells, and inflammation.^15,16,17,18^ Therefore, the aim of our study was to assess the association between adherence to the MedDiet pattern (as measured by the MedDiet score [MDS] originally proposed by Trichopoulou et al^19^ in 2005) and the risk of ORC in the EPIC cohort and to investigate the mediating role of body mass index (BMI) and waist to hip ratio (WHR) in the association.

## Methods

### Study Population

This cohort study uses data from the EPIC study, a large, multicenter, prospective cohort that enrolled 521 324 participants aged 35 to 70 years from 1992 to 2000 across 23 centers in 10 countries (Denmark, France, Germany, Greece, Italy, the Netherlands, Norway, Spain, Sweden, and the UK). Detailed methods and inclusion criteria are described elsewhere.^20,21^ At recruitment, participants completed questionnaires on diet, lifestyle, and medical history, and anthropometric measurements were collected at baseline. This study adhered to the Declaration of Helsinki^22^ and was approved by the International Agency for Research on Cancer Ethics Committee, as well as the local ethics committees of the study centers. All participants provided written informed consent for data collection and storage and individual follow-up. This report follows the Strengthening the Reporting of Observational Studies in Epidemiology (STROBE) reporting guideline for observational studies.

**Figure 1.**
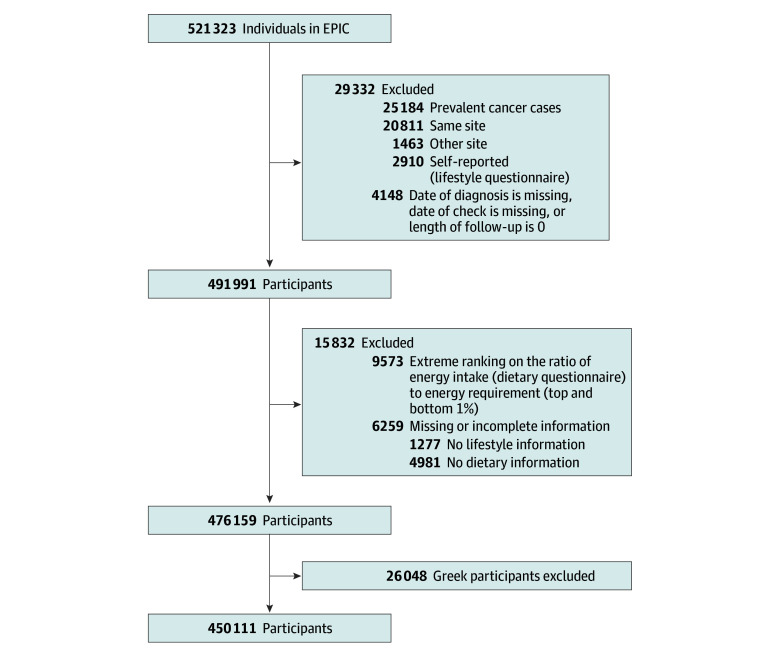
Flowchart of European Prospective Investigation Into Cancer and Nutrition (EPIC) Cohort

For this analysis, we excluded 25 184 individuals with cancer at baseline, 4148 with missing diagnosis dates or follow-up information, 6259 lacking dietary or lifestyle data, and 9573 with extreme values (top and bottom 1%) in the energy intake to requirement ratio to reduce potential measurement errors in dietary reporting. Energy requirements were estimated for each participant using estimating equations to calculate the basal metabolic rate. The basal metabolic rate was then adjusted by a physical activity factor to estimate total energy expenditure. The ratio of energy intake to energy requirement was calculated for each participant to check whether their reported intake aligned with their estimated needs. Participants with ratios outside the 1st or 99th percentiles were identified as having implausible energy intakes based on their estimated requirements. In addition, 26 048 Greek participants (5% of the overall sample) were excluded. Overall, 450 111 participants were included in the current analyses (Figure 1).

### Dietary Assessment

Diet prior to baseline was assessed at the time of recruitment using country-specific questionnaires,^23,24,25^ which were validated within each center. These assessments included the 260-item self-administered semiquantitative food-frequency questionnaire, semiquantitative food-frequency questionnaire combined with dietary record, and diet history questionnaire administered through interviews.^23^ Nutrient intakes were determined using country-specific food composition tables. In this study, we focused on 14 food groups and nutrients, namely, vegetables, legumes, fruits, nuts and seeds, dairy products, cereals, meat and meat products, fish and seafood, monounsaturated fats, polyunsaturated fats, and saturated fats. For each participant, we estimated the daily intake (in grams) of these different dietary factors, as well as total energy intake.

### Assessment of Covariates and Anthropometric Variables

To gather information on lifestyle and health, a validated questionnaire was used.^24,25^ Anthropometric measurements, including weight, height, and waist and hip circumferences, were obtained using a standard protocol,^21^ except for participants residing in Oxford (UK), France, and Norway, which collected self-reported data following a specific protocol to reduce heterogeneity due to clothing differences.^26^ For the current study, we used BMI (calculated as weight in kilograms divided by height in meters squared) as the primary indicator of general obesity, whereas WHR was used as a proxy of abdominal obesity. Information on smoking status and intensity (never smokers; current smokers of 1-15, 16-25, and >25 cigarettes/d; former smokers who quit ≤10, 11-20, and >20 years before recruitment; current smokers of cigars and pipes and occasional current smokers; current smokers with unknown intensity; and not specified), education level (according to the maximum achieved education level [primary school, technical school, secondary school, or university degree]), and physical activity (inactive, moderately inactive, moderately active, active) were used as confounders in the multivariable Cox regression model.^27^ Furthermore, to handle missing data for these variables, specifically physical activity (8824 participants [2% of the final sample]), education level (16 873 participants [3.7% of the final sample]), smoking status and intensity (8423 participants [1.9% of the final sample]) and type 2 diabetes (38 922 participants [8.6% of the final sample]), we used imputation to account for missing values.

### Appraisal of Adherence to the MedDiet

An MDS was developed by Trichopoulou et al^28^ in 1995. We used the 9-item version of the MDS adapted by Trichopoulou et al^19^ in 2005, which assesses fat intake by calculating the ratio of unsaturated (the sum of monounsaturated and polyunsaturated fats) to saturated fats. The other elements were fruit and nuts, vegetables (excluding potatoes), legumes, cereals, fish, dairy products, meat products, and alcohol. A value of 0 or 1 was assigned to each component of the score. For beneficial components that are highly consumed in Mediterranean countries (vegetables, legumes, fruits and nuts, cereals, fish, and a high ratio of unsaturated to saturated fats), participants were assigned a value of 0 if their consumption was below or equal to the country sex-specific median and a value of 1 if above. For the 2 less consumed and more detrimental components (dairy and meat and meat products), individuals were assigned a value of 1 if their consumption was below the country- and sex-specific median and a value of 0 if above. Additionally, a value of 1 was given to participants who consumed a moderate amount of alcohol (ie, 10-50 g/d of ethanol for men and 5-25 g/d for women). A value of 0 was assigned to participants who consumed other quantities of alcohol (ie, <10 or >50 g/d of ethanol for men and <5 or >25 g/d for women).^29^ Participants were grouped into the following 3 MDS categories according to their adherence to the MedDiet: low (0-3 points), medium (4-6 points), or high (7-9 points).

A variation of the MDS, the relative MedDiet score (rMedDiet), was also calculated.^10^ Briefly, this score included 9 nutritional components: 7 beneficial components (vegetables, legumes, fruit and nuts, cereals, fish and seafood, olive oil, and moderate alcohol consumption) and 2 detrimental components (meat and meat products and dairy products). Each component of the score, except for alcohol, was always measured in grams per 1000 kcal.^29^ In the rMedDiet, all components of the score, except for olive oil and alcohol, were divided into tertiles of dietary intake. The rMedDiet ranged from 0 to 18 points. Adherence to the rMedDiet was further classified into categories of low (0-6 points), medium (7-10 points), or high (11-18 points) adherence to the MedDiet. Therefore, the main difference between the MDS and the rMedDiet, apart from the scoring method, is the inclusion of olive oil in the latter.

### Ascertainment of ORC Cases

Incident cancer cases in the EPIC study were identified during the follow-up period based on population cancer registries in 7 of the participating countries (Denmark, Italy, the Netherlands, Norway, Spain, Sweden, and the UK) and a combination of methods, including health insurance records, cancer and pathology registries, and active follow-up through study participants and their next of kin. Follow-up was completed from 2008 through 2013, depending on the center. Cancers were classified using the *International Statistical Classification of Diseases, Tenth Revision* and the *International Classification of Diseases for Oncology, Second Edition*.^30^ We considered the outcome of interest to be the incidence of any of the following ORCs: esophageal adenocarcinoma, postmenopausal breast carcinoma (considered only for women who were postmenopausal at baseline), colorectal cancer, uterine cancer, gallbladder cancer, stomach cancer, kidney cancer, liver cancer, cholangiocarcinoma, ovarian cancer, pancreatic cancer, thyroid cancer, meningioma, and multiple myeloma.^4^

### Statistical Analysis

The baseline characteristics of the participants are described as mean (SD) or median (minimum-maximum) for continuous variables. The MDS was assessed as a categorical variable according to low (0-3 points), medium (4-6 points), and high (7-9 points) MedDiet adherence, using the lowest category as the reference, as well as per 1-unit increase in the score (MDS as continuous). Restricted cubic splines with 4 knots tested nonlinearity using the likelihood ratio test, and linear trends were modeled by assigning participants the median value in each MDS category. Cox proportional hazards regression models with age as the timescale were used to assess the association between MDS and ORC risk, both as a categorical variable and per 1-point MDS increase, with low adherence as the reference. Models were stratified by country, sex, and age at recruitment, adjusting for relevant covariates. The time at entry was age at recruitment, while the time at exit was age at cancer diagnosis. For participants who did not experience the event of interest, the time at exit was age at death, loss to follow-up, or end of follow-up, whichever occurred last. The latter participants were censored at the time of exit.

Additionally, we further examined associations with ORC subtypes, specifically hormone-related cancers, adjusting for reproductive variables. Stratified analyses were performed by BMI, physical activity, smoking status, sex, and education. The proportional hazards assumption was checked using Schoenfeld residuals. Additional Cox models were adjusted separately for BMI, WHR, and both to explore potential changes in results, considering these variables as potential mediators.

Sensitivity analyses were conducted using the rMedDiet, which showed high consistency (Cronbach α = 0.83; 95% CI, 0.82-0.83). We excluded the first 2 years of follow-up and reran analyses without the alcohol component in the MDS. We then assessed the potential association of each component of the MDS individually and mutually adjusting for each other. Additionally, we investigated the association between adherence to the MedDiet and different subtypes of ORC separately and with specific consideration of hormone-related cancers among women.

We also explored whether the association between adherence to the MedDiet and ORC could be partially mediated by BMI or WHR (eFigure in Supplement 1). Therefore, a mediation analysis was used to understand how much of the association of MedDiet on ORC risk could be explained by BMI or WHR as indicators of general and abdominal adiposity, respectively, using the method proposed by VanderWeele and Vansteelandt.^31,32,33^ Mediating associations were assessed separately for each of the considered mediators. Two models were specified to estimate effects and hazard ratios (HRs) for mediation, adjusting for the exposure. In the outcome model, ORC was regressed on both the mediator and the exposure using a Cox proportional hazards regression model. Each mediator was also regressed on the exposure. The total effect was calculated from a multivariable Cox model assessing MedDiet adherence and ORC risk, including an interaction between exposure and mediator. All models were adjusted for previously mentioned confounders, with exposure-mediator interactions also considered.

All analyses were performed from March 1 to May 31, 2023, using R, version 4.2.3 (R Foundation). We used the cmest function in the CMAverse R package to perform the mediation analysis.^34^ The threshold for significance was *P* < .05.

## Results

A total of 450 111 participants were included in the study (mean [SD] age, 51.1 [9.8] years; 29.2% men and 70.8% women) and followed up during a median (IQR) time of 14.9 years (4.1 years). We identified 4.9% of participants with incident ORC in the EPIC study who were initially free of cancer (rates, 0.053, 0.049, and 0.043 per person-year in the low, medium, and high MedDiet adherence groups, respectively). Baseline characteristics of participants stratified by the 3 levels of MDS are shown in Table 1. At baseline, the mean (SD) BMI was 25.3 (4.2) and WHR, 0.8 (0.1). Participants in the highest MDS category were generally younger, had a higher education level, were more likely to be never smokers, were less physically active, and showed a higher energy intake.

**Table 1.** Baseline Characteristics by Categories of the 9-Item MedDiet Score Among EPIC Study Participants

Characteristic	Participants, No. (%)			
	Low MedDiet adherence (0-3 points)	Medium MedDiet adherence (4-6 points)	High MedDiet adherence (7-9 points)	Overall EPIC cohort
No. of participants	154 463 (34.3)	177 074 (39.3)	118 574 (26.3)	450 111
Age at recruitment, y				
Mean (SD)	51.6 (9.7)	51.4 (9.5)	50.1 (10.1)	51.1 (9.8)
Median (range)	52.0 (19.9-94.7)	51.6 (20.0-97.7)	50.5 (17.8-98.5)	51.5 (17.8-98.5)
Sex				
Female	108 019 (69.9)	127 978 (72.3)	82 689 (69.7)	318 686 (70.8)
Male	46 444 (30.1)	49 096 (27.7)	35 885 (30.3)	131 425 (29.2)
Country				
France	13 760 (8.9)	30 880 (17.4)	22 763 (19.2)	67 403 (15.0)
Italy	5155 (3.3)	18 071 (10.2)	21 319 (18.0)	44 545 (9.9)
Spain	3019 (2.0)	14 879 (8.4)	22 091 (18.6)	39 989 (8.9)
UK	12 317 (8.0)	29 514 (16.7)	33 585 (28.3)	75 416 (16.8)
The Netherlands	23 513 (15.2)	11 221 (6.3)	1804 (1.5)	36 538 (8.1)
Germany	27 517 (17.8)	17 547 (9.9)	3493 (2.9)	48 557 (10.8)
Sweden	30 723 (19.9)	15 083 (8.5)	2868 (2.4)	48 674 (10.8)
Denmark	25 887 (16.8)	22 658 (12.8)	6469 (5.5)	55 014 (12.2)
Norway	12 572 (8.1)	17 221 (9.7)	4182 (3.5)	33 975 (7.5)
Height, cm				
Mean (SD)	167 (8.9)	166 (8.9)	165 (8.7)	166 (8.9)
Median (range)	167 (106-210)	165 (100-210)	165 (116-201)	165 (100-210)
BMI				
Mean (SD)	25.4 (4.2)	25.2 (4.2)	25.2 (4.2)	25.3 (4.2)
Median (range)	24.8 (10.2-77.9)	24.6 (12.7-74.5)	24.6 (13.2-67.4)	24.7 (10.2-77.9)
WHR				
Mean (SD)	0.8 (0.1)	0.8 (0.1)	0.8 (0.1)	0.8 (0.1)
Median (range)	0.8 (0.5-1.8)	0.8 (0.4-1.9)	0.8 (0.5-1.9)	0.8 (0.4-1.9)
Missing	40 590 (26.3)	44 544 (25.2)	20 746 (17.5)	105 880 (23.5)
Educational level				
None	1685 (1.1)	5849 (3.3)	8017 (6.8)	15 551 (3.5)
Primary school completed	43 022 (27.9)	42 129 (23.8)	25 913 (21.9)	111 064 (24.7)
Technical/professional school	45 212 (29.3)	39 754 (22.5)	18 816 (15.9)	103 782 (23.1)
Secondary school	29 362 (19.0)	38 267 (21.6)	26 281 (22.2)	93 910 (20.9)
Longer education	31 743 (20.6)	44 052 (24.9)	33 136 (27.9)	108 931 (24.2)
Missing	3439 (2.2)	7023 (4.0)	6411 (5.4)	16 873 (3.7)
Physical activity level^a^				
Inactive	27 689 (17.9)	34 120 (19.3)	26 223 (22.1)	88 032 (19.6)
Moderately inactive	50 365 (32.6)	59 580 (33.6)	39 996 (33.7)	149 941 (33.3)
Moderately active	41 228 (26.7)	48 371 (27.3)	30 600 (25.8)	120 199 (26.7)
Active	30 736 (19.9)	31 605 (17.8)	20 774 (17.5)	83 115 (18.5)
Missing	4445 (2.9)	3398 (1.9)	981 (0.8)	8824 (2.0)
Smoking status				
Never	70 472 (45.6)	87 195 (49.2)	61 627 (52.0)	219 294 (48.7)
Former	40 019 (25.9)	49 082 (27.7)	33 579 (28.3)	122 680 (27.3)
Smoker	41 680 (27.0)	36 969 (20.9)	21 065 (17.8)	99 714 (22.2)
Missing	2292 (1.5)	3828 (2.2)	2303 (1.9)	8423 (1.9)
Duration of smoking, y				
≤10	12 460 (8.1)	14 834 (8.4)	10 317 (8.7)	37 611 (8.4)
11-20	15 257 (9.9)	18 625 (10.5)	13 164 (11.1)	47 046 (10.5)
21-30	20 706 (13.4)	23 486 (13.3)	15 339 (12.9)	59 531 (13.2)
31-40	20 441 (13.2)	18 158 (10.3)	9738 (8.2)	48 337 (10.7)
41-50	8706 (5.6)	6037 (3.4)	2862 (2.4)	17605 (3.9)
>50	723 (0.5)	556 (0.3)	239 (0.2)	1518 (0.3)
Missing	76 170 (49.3)	95 378 (53.9)	66 915 (56.4)	238 463 (53.0)
Smoking intensity				
Never	63 540 (41.1)	75 056 (42.4)	52 807 (44.5)	191 403 (42.5)
Current, 1-15 cigarettes/d	21 942 (14.2)	19 702 (11.1)	10 796 (9.1)	52 440 (11.7)
Current, 16-25 cigarettes/d	12 579 (8.1)	9919 (5.6)	5125 (4.3)	27623 (6.1)
Current, ≥26 cigarettes/d	2831 (1.8)	2277 (1.3)	1451 (1.2)	6559 (1.5)
Former, quit ≤10 y	14 088 (9.1)	16 775 (9.5)	12 477 (10.5)	43 340 (9.6)
Former, quit 11-20 y	11 881 (7.7)	15 121 (8.5)	10 668 (9.0)	37 670 (8.4)
Former, quit ≥20 y	12 188 (7.9)	15 276 (8.6)	9381 (7.9)	36 845 (8.2)
Current, pipe, cigar, occasionally	10 496 (6.8)	16 805 (9.5)	12 606 (10.6)	39 907 (8.9)
Missing	4918 (3.2)	6143 (3.5)	3263 (2.8)	14 324 (3.2)
Alcohol intake at recruitment, g/d				
Mean (SD)	10.8 (18.1)	11.6 (16.3)	13.1 (15.6)	11.7 (16.8)
Median (range)	3.2 (0-329)	5.7 (0-300)	8.9 (0-256)	5.5 (0-329)
Total energy intake, kcal/d				
Mean (SD)	1970 (593)	2080 (624)	2210 (616)	2080 (619)
Median (range)	1890 (584-6090)	2000 (627-6450)	2140 (686-5820)	2000 (584-6450)
Age at first menstrual period, y				
Mean (SD)	13.2 (1.6)	13.0 (1.5)	12.9 (1.5)	13.1 (1.5)
Median (range)	13.0 (8.0-20.0)	13.0 (8.0-20.0)	13.0 (8.0-20.0)	13.0 (8.0-20.0)
Missing	52 883 (34.2)	52 291 (29.5)	36 983 (31.2)	142 157 (31.6)
Age at first full-term pregnancy, y				
Mean (SD)	24.6 (4.4)	24.9 (4.3)	25.3 (4.3)	24.9 (4.3)
Median (range)	24.0 (11.0-55.0)	24.0 (13.0-56.0)	25.0 (13.0-55.0)	24.0 (11.0-56.0)
Missing	67 205 (43.5)	72 049 (40.7)	53 787 (45.4)	193 041 (42.9)
Age at menopause, y				
Mean (SD)	48.7 (4.9)	48.8 (4.9)	48.6 (5.2)	48.7 (5.0)
Median (range)	50.0 (13.0-67.0)	50.0 (15.0-66.0)	50.0 (11.0-67.0)	50.0 (11.0-67.0)
Missing	113 215 (73.3)	134 049 (75.7)	93 090 (78.5)	340 354 (75.6)
Ever use of hormone replacement therapy				
No	67 526 (43.7)	87 627 (49.5)	61 641 (52.0)	216 794 (48.2)
Yes	28 372 (18.4)	32 982 (18.6)	18 928 (16.0)	80 282 (17.8)
Missing	58 565 (37.9)	56 465 (31.9)	38 005 (32.1)	153 035 (34.0)
Ever use of oral contraceptives				
No	37 353 (24.2)	50 112 (28.3)	33 338 (28.1)	120 803 (26.8)
Yes	65 262 (42.3)	75 608 (42.7)	48 585 (41.0)	189 455 (42.1)
Missing	51 848 (33.6)	51 354 (29.0)	36 651 (30.9)	139 853 (31.1)
Hysterectomy				
No	76 986 (49.8)	101 896 (57.5)	71 073 (59.9)	249 955 (55.5)
Yes	12 358 (8.0)	12 900 (7.3)	8008 (6.8)	33 266 (7.4)
Missing	65 119 (42.2)	62 278 (35.2)	39 493 (33.3)	166 890 (37.1)
Ovariectomy				
No	70 396 (45.6)	92 652 (52.3)	69 646 (58.7)	232 694 (51.7)
Yes	3830 (2.5)	3980 (2.2)	2596 (2.2)	10 406 (2.3)
Missing	80 237 (51.9)	80 442 (45.4)	46 332 (39.1)	207 011 (46.0)
Diabetes				
No	138 961 (90.0)	155 349 (87.7)	106 141 (89.5)	400 451 (89.0)
Yes	3293 (2.1)	4364 (2.5)	3081 (2.6)	10 738 (2.4)
Missing	12 209 (7.9)	17 361 (9.8)	9352 (7.9)	38 922 (8.6)

Abbreviations: BMI, body mass index (calculated as weight in kilograms divided by height in meters squared); EPIC, European Prospective Investigation Into Cancer and Nutrition; MedDiet, Mediterranean diet; WHR, waist to hip ratio.

^a^ By Cambridge Physical Activity Index.

The adjusted HRs for overall ORC according to MDS categories are shown in Table 2. Overall, higher MedDiet adherence (7-9 points vs 0-3 points) was associated with a lower risk of ORC (HR, 0.94; 95% CI, 0.90-0.98) in the fully adjusted model. Similar estimates were obtained for the association between medium adherence and ORC (*P* for trend < .001). However, no association was observed when the MDS was modeled continuously (HR, 0.94; 95% CI, 0.81-1.11).

**Table 2.** Multivariable Analysis of Incident Cases of ORC by Category of the MDS^a^ Among EPIC Study Participants

	HR (95% CI)			*P* value for trend	MDS, continuous per 1-unit increase (95% CI)
	Low MedDiet adherence (0-3 points)	Medium MedDiet adherence (4-6 points)	High MedDiet adherence (7-9 points)		
No. of participants	154 463	177 074	118 574	NA	NA
Incident cases of ORC	8255	8701	5101	NA	NA
Age-adjusted model	1 [Reference]	0.95 (0.93-0.99)	0.92 (0.89-0.97)	<.001	0.94 (0.81-1.10)
Multivariable-adjusted model^b^	1 [Reference]	0.96 (0.94-1.00)	0.94 (0.90-0.98)	<.001	0.94 (0.81-1.11)

Abbreviations: EPIC, European Prospective Investigation Into Cancer and Nutrition; MDS, Mediterranean diet score; MedDiet, Mediterranean diet; NA, not applicable; ORC, obesity-related cancer.

^a^ Score based on the traditional MedDiet was constructed by Trichopoulou et al.^19^

^b^ Model stratified by country, sex, and age at recruitment (in 1-year categories) and adjusted for attained level of education (primary school, technical school, secondary school, university degree), physical activity (inactive, moderately inactive, moderately active, active), smoking status (never smoker, former smoker, current smoker) and intensity at recruitment, height, alcohol intake at baseline (grams per day), total energy intake (kilocalories per day), and history of type 2 diabetes (yes, no).

**Figure 2.**
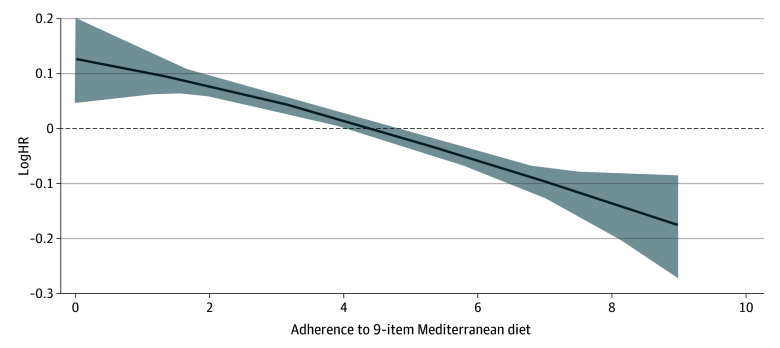
Assessment of Linear Associations Between Mediterranean Diet Score and Incident Obesity-Related Cancer (ORC) Using Restricted Cubic Splines The *P* values were obtained by testing for nonlinearity using a likelihood ratio test comparing 2 multivariable nested models: 1 model with only a linear term and 1 with a linear term and restricted cubic spline terms (*P* = .79). The reported values are in terms of the logarithmic hazard ratio (logHR), which can be converted to HR using the equation HR = exp(logHR). For example, the logHRs of 0.20, −0.20, and −0.30 correspond to HRs of 1.22, 0.82, and 0.74, respectively. In addition, logHRs of 0.10, −0.05, and −0.10 correspond to HRs of 1.10, 0.95, and 0.90, respectively. The fully adjusted model was stratified by country and sex and adjusted for age at recruitment (in 1-year categories), attained level of education (primary school, technical school, secondary school, university degree), physical activity (inactive, moderately inactive, moderately active, active), smoking status (never smoker, former smoker, current smoker) and intensity at recruitment, height, alcohol intake at recruitment (in grams per day), total energy intake at recruitment (kilocalories per day), and history of type 2 diabetes (yes, no).

We also assessed the association of the MDS with ORC using restricted cubic splines. We found no significant departure from linearity in the fully adjusted model (Figure 2).

Furthermore, our sensitivity analyses using the rMedDiet yielded comparable findings. Specifically, we observed similar results (high vs low MDS: HR, 0.93; 95% CI, 0.89-0.98) in the multivariable-adjusted model (*P* for trend < .001) (eTable 1 in Supplement 1). When we excluded the alcohol component from the original score and adjusted our analyses for alcohol intake, we obtained similar estimates (high vs low MDS: HR, 0.94; 95% CI, 0.90-0.99; *P* for trend = .06) (eTable 2 in Supplement 1). Moreover, when we excluded the first 2 years of follow-up, we obtained consistent findings (high vs low MDS: HR, 0.94; 95% CI, 0.90-0.98) (eTable 3 in Supplement 1).

When examining site-specific ORCs, we observed an inverse association between higher adherence to the MedDiet and the risk of colorectal (HR, 0.92; 95% CI, 0.85-0.99), hepatocellular (HR, 0.52; 95% CI, 0.33-0.83), and kidney (HR, 0.67; 95% CI, 0.55-0.82) cancers. Medium adherence to the MedDiet was inversely associated with esophageal cancer (HR, 0.66; 95% CI, 0.48-0.93), but not statistically significant for multiple myeloma (HR, 0.90; 95% CI, 0.81-1.01) (eTable 4 in Supplement 1). For hormone-related cancers in women, specifically postmenopausal breast cancer, endometrial cancer, and ovarian cancer, no associations were observed (eTable 5 in Supplement 1). In stratified analyses (eTable 6 in Supplement 1), we observed significant interactions by smoking status and sex in the fully adjusted model. Specifically, adherence to the MDS was inversely associated with the risk of ORC in former smokers (medium adherence: HR, 0.93 [95% CI 0.90-0.97]; high adherence: HR, 0.91 [95% CI, 0.85-0.98]) and current smokers (high adherence: HR, 0.86; 95% CI, 0.80-0.94; *P* for interaction = .04). When analyzing men and women separately, only medium vs low adherence to the MedDiet was associated with lower ORC risk (men: HR, 0.93 [95% CI, 0.89-0.97]; women: HR, 0.97 [95% CI, 0.95-1.00]; *P* for interaction = .01) (eTable 6 in Supplement 1). Results from the assessment of each individual component of the MedDiet with incident ORC is shown in eTable 7 in Supplement 1. Generally, we found a lower risk of incident ORC for moderate intake of alcohol and lower meat consumption. Finally, we present results from the exploration of the associations between each mediator and the outcome (eTables 8 and 9 in Supplement 1). Our mediation analysis did not show any significant result when considering high vs low adherence to the MedDiet and the risk of ORC mediated by BMI or WHR (eTable 10 in Supplement 1).

## Discussion

This cohort study found that greater adherence to the MedDiet was associated with a modestly reduced risk of 6% of ORCs within the EPIC study, which includes both Mediterranean and non-Mediterranean populations. Our results align with the Netherlands Cohort Study,^35^ which found an inverse association between an alternate MDS (excluding alcohol) and overall cancer incidence. Additionally, a prior EPIC analysis reported lower overall cancer risk associated with increased MedDiet adherence per 2-point increase in the MDS (HR, 0.96; 95% CI, 0.95-0.98).^11^ Adherence to the MedDiet has been linked with reduced central adiposity,^36,37,38,39^ lower BMI, and less weight gain, supporting our hypothesis that the association between MedDiet adherence and reduced ORC risk may be associated with BMI and WHR. However, our findings suggest that the observed protective association with ORCs may involve other mechanisms. For example, a previous EPIC cohort study in Italy found that the protective association of the MedDiet with colorectal cancer was not mediated by abdominal adiposity.^14^

Intervention studies have also shown that the MedDiet is positively associated with metabolic and inflammatory markers, such as fasting blood glucose and C-reactive protein.^40^ On the other hand, fiber may counteract carcinogenic N-nitroso compounds from processed meats and other sources.^41,42^ As for site-specific ORCs, we found that higher MedDiet adherence was inversely associated with the risk of colorectal, hepatocellular, and kidney cancers, while medium adherence was associated with lower esophageal cancer and multiple myeloma risks.^43^ These results align with previous studies on hepatocellular cancer,^44^ colorectal cancer,^14,45^ and esophageal adenocarcinoma.^46^ The potential benefits of the MedDiet for cancer prevention may be from interactions and synergistic effects among its various components, collectively enhancing health benefits beyond those observed for individual foods alone.^47^ Our findings suggest that higher cereal and lower meat consumption may be linked to a slightly reduced risk of ORCs. Notably, red and processed meat have been consistently associated with cancer risk.^48^ On the other hand, stronger protective associations were observed among smokers, suggesting that adherence to the MedDiet may partially offset the influence of tobacco on cancer, aligning with previous studies^49^ that found a combined association of smoking and poor MedDiet adherence with increased cancer-related mortality. Our mediation analysis did not show WHR or BMI as mediators between MedDiet and ORC risk, possibly due to the low prevalence of obesity in our cohort and the distinct contribution of general vs abdominal obesity to metabolic disruptions. Future studies should include repeated measures of exposure and mediators to explore these comparisons further.

### Strengths and Limitations

The strengths of our study include a large sample, a substantial number of cancer cases, and an extended follow-up period. Unlike prior EPIC analyses, ours assessed various cancer subtypes and obesity-cancer associations in a population encompassing diverse levels of MedDiet adherence from Mediterranean and non-Mediterranean countries. Additionally, sensitivity analyses and adjustments for smoking and other potential confounders enhance the robustness of our findings.

We also acknowledge several limitations in our study. First, exposure and potential confounders were assessed only at baseline. Although changes in diet or confounding factors may have occurred during follow-up, previous research in similar cohorts suggested that dietary patterns tend to remain relatively stable over time, which partially mitigates this limitation.^50^ Second, the Mediterranean lifestyle pattern may not be fully captured by the assessed scores, especially since a substantial portion of participants were from non-Mediterranean countries. However, in prospective studies, any misclassification within the scoring system may bias HR estimates toward the null. Third, a potential drawback of the MDS is that it treats all components with the same level of importance and simply indicates whether the consumption of each component is either above or below a designated cutoff. Additionally, the use of self-reported anthropometric measures for participants in France and Norway may have introduced bias. Nonetheless, previous studies have reported that such biases tend to be modest and generally do not substantially influence the associations with health outcomes.^50,51,52,53,54,55,56^ Future research should incorporate more rigorous validation of self-reported data or use objective measurements to address this issue.^57,58,59^ Finally, the low prevalence of overweight and obesity in our cohort may also partly explain the null results in our mediation analyses.

## Conclusions

The findings of this cohort study indicate that higher adherence to the MedDiet may slightly reduce the risk of ORCs. In addition, we observed that even a medium adherence was associated with a small reduction in the risk of these specific cancers. Although our results did not suggest mediation through overweight and obesity, more studies are needed to better understand the mechanisms through which higher adherence to the MedDiet might potentially reduce cancer risk.

## Notes

Supplement 1. **eTable 1. **Sensitivity Analysis for Multivariable Hazard Ratios (HRs) and 95% CIs of 22 057 Incident Obesity-Related Cancers According to Categories of Adherence to the 18-Item Relative Mediterranean Diet Score Among 450 111 Participants in EPIC Study **eTable 2. **Sensitivity Analysis for Multivariable Hazard Ratios (HRs) and 95% CIs of 22 057 Incident Obesity-Related Cancers According to Categories of Adherence to the Modified Mediterranean Diet Score Without the Alcohol Component Among 450 111 Participants in the EPIC Study **eTable 3. **Multivariable Hazard Ratios (HRs) and 95% CIs of 19 984 Incident Obesity-Related Cancers According to Categories of Adherence to the Modified Mediterranean Diet Score Among 442 436 Participants in EPIC Study After Excluding Participants During the First 2 Years of Follow-Up **eTable 4. **Multivariable Hazard Ratios (HRs) and 95% CIs of Site-Specific Incident Obesity-Related Cancers Subtypes According to Categories of Adherence to the Modified Mediterranean Diet in the Whole Cohort of EPIC Study **eTable 5. **Multivariable Hazard Ratios (HRs) and 95% CIs of Incident Hormone-Related Cancers Subtypes According to Categories of Adherence to the Modified Mediterranean Diet Among Females Within EPIC Study **eTable 6.** Stratified Analyses Considering Potential Effect Modifiers: Multivariable Hazard Ratios (HRs) and 95% CIs of 22 057 Incident Obesity-Related Cancers According to Categories of Adherence to Modified Mediterranean Diet Score Among 450 111 Participants in EPIC Study **eTable 7. **Multivariable Hazard Ratios (HRs) and 95% CIs of 22 057 Incident Obesity-Related Cancers According to Sex-Specific Median Intake of Each Component of the Mediterranean Diet Score Among 450 111 Participants in EPIC Study **eTable 8. **Explored Associations Between Adherence to the Mediterranean Diet (Categorized as High vs Low Adherence) and BMI and WHR Continuous Mediators and These Mediators With Incident ORC in the EPIC Study **eTable 9.** Explored Associations Between Adherence to the Mediterranean Diet (Categorized as Medium vs Low Adherence) and BMI and WHR Continuous Mediators and These Mediators With Incident ORC in the EPIC Study **eTable 10. **Results From Mediation Analyses for Exploring Mediating Effects Between Adherence to the Mediterranean Diet Score (High vs low adherence) and Obesity-Related Cancers (ORCs) by Baseline Continuous Mediators: BMI and WHR in the EPIC Study **eFigure. **DAGs for Exploring Whether the Association Between Mediterranean diet score and ORC Could Be Partially Mediated by Body Mass Index Within the EPIC Cohort Study

Supplement 2.
Data Sharing Statement
