# Supplementary material for: Adherence to the Mediterranean Diet and Obesity-Linked Cancer Risk in EPIC
Source: JAMA Netw Open. 2025 Feb 25;8(2):e2461031. doi: 10.1001/jamanetworkopen.2024.61031 (PMC11862969; doi:10.1001/jamanetworkopen.2024.61031)
Supplement: Supplement 1. — eTable 1. Sensitivity Analysis for Multivariable Hazard Ratios (HRs) and 95% CIs of 22 057 Incident Obesity-Related Cancers According to Categories of Adherence to the 18-Item Relative Mediterranean Diet Score Among 450 111 Participants in EPIC Study eTable 2. Sensitivity Analysis for Multivariable Hazard Ratios (HRs) and 95% CIs of 22 057 Incident Obesity-Related Cancers According to Categories of Adherence to the Modified Mediterranean Diet Score Without the Alcohol Component Among 450 111 Participants in the EPIC Study eTable 3. Multivariable Hazard Ratios (HRs) and 95% CIs of 19 984 Incident Obesity-Related Cancers According to Categories of Adherence to the Modified Mediterranean Diet Score Among 442 436 Participants in EPIC Study After Excluding Participants During the First 2 Years of Follow-Up eTable 4. Multivariable Hazard Ratios (HRs) and 95% CIs of Site-Specific Incident Obesity-Related Cancers Subtypes According to Categories of Adherence to the Modified Mediterranean Diet in the Whole Cohort of EPIC Study eTable 5. Multivariable Hazard Ratios (HRs) and 95% CIs of Incident Hormone-Related Cancers Subtypes According to Categories of Adherence to the Modified Mediterranean Diet Among Females Within EPIC Study eTable 6. Stratified Analyses Considering Potential Effect Modifiers: Multivariable Hazard Ratios (HRs) and 95% CIs of 22 057 Incident Obesity-Related Cancers According to Categories of Adherence to Modified Mediterranean Diet Score Among 450 111 Participants in EPIC Study eTable 7. Multivariable Hazard Ratios (HRs) and 95% CIs of 22 057 Incident Obesity-Related Cancers According to Sex-Specific Median Intake of Each Component of the Mediterranean Diet Score Among 450 111 Participants in EPIC Study eTable 8. Explored Associations Between Adherence to the Mediterranean Diet (Categorized as High vs Low Adherence) and BMI and WHR Continuous Mediators and These Mediators With Incident ORC in the EPIC Study eTable 9. Explored Associations Between Adherence [file jamanetwopen-e2461031-s001.pdf]

Supplemental Online Content

Aguilera-Buenosvinos I, Morales Bernstein F, González-Gil EM, et al. Adherence to Mediterranean diet and obesity-related cancer risk in EPIC. *JAMA Netw Open*. 2025;8(2):e2461031. doi:10.1001/jamanetworkopen.2024.61031

eTable 1. Sensitivity Analysis for Multivariable Hazard Ratios (HRs) and 95% CIs of 22 057 Incident Obesity-Related Cancers According to Categories of Adherence to the 18-Item Relative Mediterranean Diet Score Among 450 111 Participants in EPIC Study

eTable 2. Sensitivity Analysis for Multivariable Hazard Ratios (HRs) and 95% CIs of 22 057 Incident Obesity-Related Cancers According to Categories of Adherence to the Modified Mediterranean Diet Score Without the Alcohol Component Among 450 111 Participants in the EPIC Study

eTable 3. Multivariable Hazard Ratios (HRs) and 95% CIs of 19 984 Incident Obesity-Related Cancers According to Categories of Adherence to the Modified Mediterranean Diet Score Among 442 436 Participants in EPIC Study After Excluding Participants During the First 2 Years of Follow-Up

eTable 4. Multivariable Hazard Ratios (HRs) and 95% CIs of Site-Specific Incident Obesity-Related Cancers Subtypes According to Categories of Adherence to the Modified Mediterranean Diet in the Whole Cohort of EPIC Study

eTable 5. Multivariable Hazard Ratios (HRs) and 95% CIs of Incident Hormone-Related Cancers Subtypes According to Categories of Adherence to the Modified Mediterranean Diet Among Females Within EPIC Study

eTable 6. Stratified Analyses Considering Potential Effect Modifiers: Multivariable Hazard Ratios (HRs) and 95% CIs of 22 057 Incident Obesity-Related Cancers According to Categories of Adherence to Modified Mediterranean Diet Score Among 450 111 Participants in EPIC Study

eTable 7. Multivariable Hazard Ratios (HRs) and 95% CIs of 22 057 Incident Obesity-Related Cancers According to Sex-Specific Median Intake of Each Component of the Mediterranean Diet Score Among 450 111 Participants in EPIC Study

eTable 8. Explored Associations Between Adherence to the Mediterranean Diet (Categorized as High vs Low Adherence) and BMI and WHR Continuous Mediators and These Mediators With Incident ORC in the EPIC Study

eTable 9. Explored Associations Between Adherence to the Mediterranean Diet (Categorized as Medium vs Low Adherence) and BMI and WHR Continuous Mediators and These Mediators With Incident ORC in the EPIC Study

eTable 10. Results From Mediation Analyses for Exploring Mediating Effects Between Adherence to the Mediterranean Diet Score (High vs low adherence) and Obesity-Related Cancers (ORCs) by Baseline Continuous Mediators: BMI and WHR in the EPIC Study

eFigure. DAGs for Exploring Whether the Association Between Mediterranean diet score and ORC Could Be Partially Mediated by Body Mass Index Within the EPIC Cohort Study

This supplemental material has been provided by the authors to give readers additional information about their work.

© 2025 Aguilera-Buenosvinos I et al. *JAMA Network Open.*

Supplemental Material

eTable 1. Sensitivity Analysis for Multivariable Hazard Ratios (HRs) and 95% CIs of 22 057 Incident Obesity-Related Cancers According to Categories of Adherence to the 18-Item Relative Mediterranean Diet Score Among 450 111 Participants in EPIC Study

	**Categories of adherence of the relative Mediterranean Diet score [1]**			
	Low	Medium	High	*P for trend*
	0-6 points	7-10 points	11-18 points	
N	127,445	206,421	116,245	
Cases	6809	10252	4996	
Age-adjusted model	1 [reference]	0.94 (0.92-0.98)	0.91 (0.88-0.96)	**P < 0.001**.
Multivariable adjusted model [2]	1 [reference]	**0.96 (0.93-0.99)**	**0.93 (0.89-0.98)**	**P < 0.001**.

[1]Score modified by Romaguera et al., 2009. Categories of Mediterranean Diet score: Low adherence, 0-6; Medium adherence, 7-10; High adherence, 11-18. *Score with olive oil intake.

[2]Model stratified by country, sex, and age at recruitment (in 1-year categories), and adjusted for attained level of education (primary school, technical school, secondary school, or university degree), physical activity (inactive, moderately inactive, moderately active, and active), smoking status (never smoker, former smoker, and current smoker) and intensity at recruitment, height, alcohol intake at baseline (g/day), total energy intake (Kcal) and history of T2D (yes/no).

© 2025 Aguilera-Buenosvinos I et al. *JAMA Network Open.*

eTable 2. Sensitivity Analysis for Multivariable Hazard Ratios (HRs) and 95% CIs of 22 057 Incident Obesity-Related Cancers According to Categories of Adherence to the Modified Mediterranean Diet Score Without the Alcohol Component Among 450 111 Participants in the EPIC Study

	**Categories of adherence of the modified Mediterranean Diet score without alcohol component [1]**			
	Low	Medium	High	*P for trend*
	0-2 points	3-5 points	6-8 points	
N	100,441	263,853	85,817	
Cases	5456	12,994	3607	
Age-adjusted model	1 [reference]	0.96 (0.94-0.99)	0.92 (0.88-0.97)	0.01
Multivariable adjusted model [2]	1 [reference]	0.97 (0.95-1.00)	**0.94 (0.90-0.99)**	**0.06**

[1]Score modified to remove the alcohol component - based on the traditional Mediterranean diet has been constructed by Trichopoulou et al. (2005).
[2]Model stratified by country, sex, and age at recruitment (in 1-year categories), and adjuster for attained level of education (primary school, technical school, secondary school, or university degree), physical activity (inactive, moderately inactive, moderately active, and active), smoking status (never smoker, former smoker, and current smoker) and intensity at recruitment, height, alcohol intake at baseline (g/day), total energy intake (Kcal) and history of T2D (yes/no).

© 2025 Aguilera-Buenosvinos I et al. *JAMA Network Open.*

eTable 3. Multivariable Hazard Ratios (HRs) and 95% CIs of 19 984 Incident Obesity-Related Cancers According to Categories of Adherence to the Modified Mediterranean Diet Score Among 442 436 Participants in EPIC Study After Excluding Participants During the First 2 Years of Follow-Up

	Categories of adherence of the modified Mediterranean diet score [1]		
	Low	Medium	High
	0-3 points	4-6 points	7-9 points
N	144083	166238	112202
Cases	7473	7857	4654
Age-adjusted model	1 [reference]	**0.95 (0.93-0.99)**	**0.93 (0.89-0.97)**
Multivariable adjusted model [2]	1 [reference]	**0.96 (0.93-1.00)**	**0.94 (0.90-0.98)**

[1]Score based on the traditional Mediterranean diet has been constructed by Trichopoulou et al. (2005) -.
[2]Model stratified by country, sex, and age at recruitment (in 1-year categories), and adjusted for attained level of education (primary school, technical school, secondary school, or university degree), physical activity (inactive, moderately inactive, moderately active, and active), smoking status (never smoker, former smoker, and current smoker) and intensity at recruitment, height, alcohol intake at baseline (g/day), total energy intake (Kcal) and history of T2D (yes/no).

© 2025 Aguilera-Buenosvinos I et al. *JAMA Network Open.*

eTable 4. Multivariable Hazard Ratios (HRs) and 95% CIs of Site-Specific Incident Obesity-Related Cancers Subtypes According to Categories of Adherence to the Modified Mediterranean Diet in the Whole Cohort of EPIC Study

**Site-specific incident ORCs**	**Adherence to the modified Mediterranean diet score[1]**			
**Esophagus cancer**	Low	Medium	High	***P for trend***
N=215	0-3 points	4-6 points	7-9 points	
N	151,479	174,046	116,815	
Cases	86	60	50	
Age-adjusted model	1 [reference]	0.63 (0.46-0.88)	0.77 (0.52-1.15)	0.006
Multivariable adj. model [2]	1 [reference]	**0.66 (0.48-0.93)**	0.83 (0.56-1.25)	0.38
**Gastric cardia cancer**				
N=239				
N	151,467	174,021	116,827	
Cases	98	85	38	
Age-adjusted model	1 [reference]	0.79 (0.59-1.07)	0.61 (0.41-0.94)	0.02
Multivariable adj.model [2]	1 [reference]	0.86 (0.64-1.16)	0.71 (0.46-1.16)	0.11
**Colorectal cancer**				
N=6155				
N	149,382	171,944	115,514	
Cases	2183	2162	1351	
Age-adjusted model	1 [reference]	0.92 (0.87-0.98)	0.90 (0.83-0.98)	**0.01**
Multivariable adj.model [2]	1 [reference]	**0.93 (0.88-0.99)**	**0.92 (0.85-0.99)**	**0.03**

© 2025 Aguilera-Buenosvinos I et al. *JAMA Network Open.*

**Hepatocellular cancer**				
N=215				
N	151,479	174,034	116,827	
Cases	86	72	38	
Age-adjusted model	1 [reference]	0.70 (0.51-0.96)	**0.49 (0.31-0.78)**	**0.002**
Multivariable adj.model [2]	1 [reference]	**0.72 (0.53-1.00)**	**0.52 (0.33-0.83)**	**0.006**
**Gallbladder cancer**				
N=335				
N	151,459	173,978	116,796	
Cases	106	128	69	
Age-adjusted model	1 [reference]	1.23 (0.95-1.59)	1.03 (0.73-1.48)	0.83
Multivariable adj.model [2]	1 [reference]	1.26 (0.76-1.64)	1.07 (0.75-1.54)	0.67
**Pancreatic cancer**				
N=1289				
N	151,077	173,661	116,593	
Cases	488	445	272	
Age-adjusted model	1 [reference]	0.88 (0.77-0.99)	0.87 (0.73-1.04)	0.12
Multivariable adj.model [2]	1 [reference]	0.92 (0.81-1.06)	0.94 (0.79-1.13)	0.52
**Multiple Myeloma**				
N=1839				
N	150,915	173,502	116,444	
Cases	650	604	421	
Age-adjusted model	1 [reference]	0.90 (0.81-1.01)	0.94 (0.82-1.09)	0.44

© 2025 Aguilera-Buenosvinos I et al. *JAMA Network Open.*

Multivariable adj.model [2]	1 [reference]	**0.89 (0.80-0.99)**	0.92 (0.80-1.07)	0.28
**Kidney cancer**				
N=1007				
N	151,182	173,778	116,676	
Cases	383	328	189	
Age-adjusted model	1 [reference]	**0.76 (0.66-0.89)**	**0.62 (0.51-0.77)**	**P < 0.001.**
Multivariable adj.model [2]	1 [reference]	**0.79 (0.69-0.93)**	**0.67 (0.55-0.82)**	**0.001**
**Thyroid cancer**				
N=759				
N	151,354	173,825	116,680	
Cases	211	281	185	
Age-adjusted model	1 [reference]	0.91 (0.76-1.10)	0.86 (0.70-1.10)	0.18
Multivariable adj.model [2]	1 [reference]	0.92 (0.77-1.12)	0.88 (0.71-1.10)	0.66

[1]Score modified to remove the alcohol component - based on the traditional Mediterranean diet proposed by Trichopoulou et al. (2005).
[2]Model stratified by country and sex, and age at recruitment (in 1-year categories), and adjuster for attained level of education (primary school, technical school, secondary school, or university degree), physical activity (inactive, moderately inactive, moderately active, and active), smoking status (never smoker, former smoker, and current smoker) and intensity at recruitment, height, alcohol intake at baseline (g/day), total energy intake (kcal/day) and prevalence of diabetes (yes/no).

© 2025 Aguilera-Buenosvinos I et al. *JAMA Network Open.*

eTable 5. Multivariable Hazard Ratios (HRs) and 95% CIs of Incident Hormone-Related Cancers Subtypes According to Categories of Adherence to the Modified Mediterranean Diet Among Females Within EPIC Study

	Adherence to the modified Mediterranean diet score[1]			
**Postmenopausal breast cancer**	Low	Medium	High	***P for trend***
n= 6633	0-3 points	4-6 points	7-9 points	
N	105,614	125,235	81,204	
Cases	2405	2743	1485	
Age-adjusted model	1 [reference]	1.04 (0.99-1.11)	1.01 (0.94-1.09)	0.79
Multivariable adjusted model 2[2]	1 [reference]	1.04 (0.99-1.11)	1.00 (0.93-1.08)	0.87
Multivariable adjusted model 3[3]	1 [reference]	1.03 (0.99-1.11)	1.00 (0.93-1.08)	0.88
**Endometrial cancer**				
n=1932				
N	107,331	127,191	82,232	
Cases	688	787	457	
Age-adjusted model	1 [reference]	1.01 (0.91-1.13)	1.02 (0.89-1.17)	0.73
Multivariable adjusted model 2[2]	1 [reference]	1.07 (0.90-1.12)	1.01 (0.89-1.17)	0.79
Multivariable adjusted model 3[3]	1 [reference]	1.00 (0.90-1.12)	1.01 (0.88-1.17)	0.83
**Ovary cancer**				
n=1415				
N	107,530	127,387	82,354	
Cases	489	591	335	

© 2025 Aguilera-Buenosvinos I et al. *JAMA Network Open.*

Age-adjusted model	1 [reference]	1.06 (0.94-1.21)	0.99 (0.85-1.17)	0.98
Multivariable adjusted model 2[2]	1 [reference]	1.07(0.94-1.22)	1.01 (0.86-1.19)	0.89
Multivariable adjusted model 3[3]	1 [reference]	1.07(0.94-1.22)	1.00 (0.86-1.19)	0.90

[1]Score based on the traditional Mediterranean diet proposed by Trichopoulou et al. (2005).

[2]Model stratified by country and sex, and age at recruitment (in 1-year categories), and adjuster for attained level of education (primary school, technical school, secondary school, or university degree), physical activity (inactive, moderately inactive, moderately active, and active), smoking status (never smoker, former smoker, and current smoker) and intensity at recruitment, height, alcohol intake (g/day) and total energy intake (Kcal/day). [3]Same model as above additionally adjuster for prevalence of diabetes (yes/no) and specific reproductive variables such as: age at first menstrual period (continuous), number of full-term pregnancies (0/1/2/≥ 3), age at first full-term pregnancy (never pregnant/quartiles), menopausal status (premenopausal, postmenopausal, surgical postmenopausal and perimenopausal), (ever use of oral contraceptive (yes/no), ever use of hormonal therapy (yes/no/missing), ovariectomy (no, unilateral, bilateral, unilateral or bilateral), and hysterectomy (no/yes).

eTable 6. Stratified Analyses Considering Potential Effect Modifiers: Multivariable Hazard Ratios (HRs) and 95% CIs of 22 057 Incident Obesity-Related Cancers According to Categories of Adherence to Modified Mediterranean Diet Score Among 450 111 Participants in EPIC Study

Interaction with smoking status[2]	Adherence to 9-item modified Mediterranean diet score[1]	N total	N events	HR	95% CI	P for interaction
Never smokers	Medium adherence (4-6 points)	227,706	10,861	0.97	0.95-1.00	
	Highest adherence (7-9 points)	227,706	10,861	0.97	0.92-1.02	
Former smokers	Medium adherence (4-6 points)	122,668	6,295	**0.93**	**0.90-0.97**	
	Highest adherence (7-9 points)	122,668	6,295	**0.91**	**0.85-0.98**	

© 2025 Aguilera-Buenosvinos I et al. *JAMA Network Open.*

Current smokers	Medium adherence (4-6 points)	99,707	4,901	0.99	0.95-1.04	
	Highest adherence (7-9 points)	99,707	4,901	**0.86**	**0.80-0.94**	
						**0.04**
**Interaction with sex[2]**						
Males	Medium adherence (4-6 points)	131,395	5,315	**0.93**	**0.89-0.97**	
	Highest Adherence (7-9 points)	131,395	5,315	1.12	0.68-1.83	
Females	Medium adherence (4-6 points)	318,686	16,742	**0.97**	**0.95-1.00**	
	Highest Adherence (7-9 points)	318,686	16,742	0.84	0.54-1.33	
						**0.01**

[1]Score based on the traditional Mediterranean diet proposed by Trichopoulou et al. (2005)[433].

[2]Model stratified by country and sex, and age at recruitment (in 1-year categories), and adjuster for, attained level of education (primary school, technical school, secondary school, or university degree), physical activity (inactive, moderately inactive, moderately active, and active), smoking status(never smoker, former smoker, and current smoker) and intensity at recruitment , height, alcohol intake at recruitment (g/day), total energy intake (Kcal/day) and history of T2D (yes/no).

© 2025 Aguilera-Buenosvinos I et al. *JAMA Network Open.*

eTable 7. Multivariable Hazard Ratios (HRs) and 95% CIs of 22 057 Incident Obesity-Related Cancers According to Sex-Specific Median Intake of Each Component of the Mediterranean Diet Score Among 450 111 Participants in EPIC Study

	Component from the Mediterranean Diet Score (sex specific medians)[1]	N per category	N events per category	HR	95% CI	*P-value*
**Alcohol component (sex-specific median intakes)[2]**						
Multivariable adjusted model[3]	**1 point:**<br>>10 g/day & ≤ 50 g/day for men<br>>5 g/day & ≤ 25 g/day for women	450,111	22,057	0.98	0.91-0.97	0.094
**Cereals Component (sex-specific median intakes)[4]**						
Multivariable adjusted model[3]	**1 point**<br>≥ 235 g/day for men<br>≥ 187 g/day for women	450,111	22,057	**0.94**	**0.91-0.97**	**0.001**
**Dairy products component (sex-specific median intakes) (reverse scoring)**						
Multivariable adjusted model[3]	**1 point**<br>< 285 g/day for men<br>< 284 g/day for women	450,111	22,057	1.01	0.98-1.04	0.624
**Fat component defined as: MUFA+PUFA/SAF ratio component (sex-specific median intakes)**						
Multivariable adjusted model[3]	**1 point**<br>≥ 1.35 g/day for men<br>≥ 1.32 g/day for women	450,111	22,057	1.01	0.98-1.04	0.528
**Fish and seafood component (sex-specific median intakes)**						

© 2025 Aguilera-Buenosvinos I et al. *JAMA Network Open.*

Multivariable adjusted model[3]	**1 point**<br>≥ 28.5 g/day for men<br>≥ 28.7 g/day for women	450,111	22,057	0.99	0.96-1.02	0.607
**Fruit and nuts component (sex-specific median intakes)**						
Multivariable adjusted model[3]	**1 point**<br>≥ 151.3 g/day for men<br>≥ 210 g/day for women	450,111	22,057	0.98	0.95-1.01	0.254
**Legumes component (sex-specific median intakes)**						
Multivariable adjusted model[3]	**1 point**<br>≥ 4.48 g/day for men<br>≥ 5.40 g/day for women	450,111	22,057	1.03	0.99-1.06	0.18
**Meat component (sex-specific median intakes) (reverse scoring)**						
Multivariable adjusted model[3]	**1 point**<br>< 121.2 g/day for men<br>< 84.22 g/day for women	450,111	22,057	**0.95**	**0.92-0.98**	**0.001**
**Vegetable component (sex-specific median intakes)**						
Multivariable adjusted model[3]	**1 point**<br>≥ 141 g/day for men<br>≥ 179 g/day for women	450,111	22,057	0.98	0.95-1.01	

[1]Score based on the traditional Mediterranean diet proposed by Trichopoulou et al. (2005)[433].
Values of zero or one were assigned to each of nine indicated components, using as cut-off values the sex specific medians among the EPIC participants. 0 points corresponds to the reference category for the Cox regression models.
[2] For alcohol component, sex-specific medians as a cut-points are based on categories of specific advice regarding alcohol consumption.
[3]Model stratified by country, sex, age at recruitment (in 1-year categories) and adjusted for attained level of education (primary school, technical school, secondary school, or university degree), physical activity (inactive, moderately inactive, moderately active, and active), smoking status (never smoker, former smoker, and current smoker) and intensity at recruitment, height, alcohol intake (g/day) and total energy intake (kcal/day) and prevalence of diabetes (yes/no). Fully multivariable Cox model was

© 2025 Aguilera-Buenosvinos I et al. *JAMA Network Open.*

mutually adjusted for the other components of the Mediterranean Diet score.[4] For cereals components, all types of refined and whole grain cereals were considered. MUFA: Monounsaturated fatty acid; PUFA: polyunsaturated fatty acid; SFA: saturated fatty acid.

eTable 8. Explored Associations Between Adherence to the Mediterranean Diet (Categorized as High vs Low Adherence) and BMI and WHR Continuous Mediators and These Mediators With Incident ORC in the EPIC Study

**Associations between modified Mediterranean diet score (high adherence vs low adherence) and each mediator**				
**Exposure[1]**	**Mediator as continuous**	**Coefficient**	**95% CI**	**P-value**
MMed score as categorical (High adherence)	Waist-to-hip ratio	-0.003	-0.005, -0.002	<0.001
MMed score as categorical (High adherence)	Body mass index	-0.20	-0.23, -0.17	<0.001

**Fully multivariable adjusted model[2]**	**Mediator as continuous**	**HR**	**95% CI**	**P-value**
Cox regression w/continuous mediator	Waist-to-hip ratio	3.02	2.41-3.81	<0.001
Cox regression w/continuous mediator	Body mass index	1.02	1.02-1.03	<0.001

**Multivariable association between the exposure and the outcome to calculate de control direct effect (CDE) adjusting for each mediator.**					
**Fully multivariable adjusted model**	**Exposure[1]**	**Mediators as continuous**	**HR**	**95% CI**	***P-value***

© 2025 Aguilera-Buenosvinos I et al. *JAMA Network Open.*

Cox regression adjusted for WHR at baseline	MMed score as categorical (High adherence)	Waist-to-hip ratio	0.96	0.94-0.99	0.049
Cox regression adjusted for BMI at baseline	MMed score as categorical (High adherence)	Body mass index	0.96	0.94-0.99	0.048

[1]Score based on the traditional Mediterranean diet score proposed by Trichopoulou et al. (2005). [2]Multivariable adjusted model for the fully multivariable model stratified by country, sex, and age at recruitment (in 1-year categories), and adjusted for attained level of education (primary school, technical school, secondary school, or university degree), physical activity (inactive, moderately inactive, moderately active, and active), smoking status(never smoker, former smoker, and current smoker) and intensity at recruitment , height, alcohol intake (g/day) and total energy intake (Kcal/day) and prevalence of diabetes (yes/no).

eTable 9. Explored Associations Between Adherence to the Mediterranean Diet (Categorized as Medium vs Low Adherence) and BMI and WHR Continuous Mediators and These Mediators With Incident ORC in the EPIC Study

**Associations between modified Mediterranean diet score (medium adherence vs low adherence) and each mediator**				
**Exposure[1]**	**Mediators as continuous**	**Coefficient**	**95% CI**	P-value
MMed score as categorical (Medium adherence)	Waist-to-hip ratio	-0.002	-0.004, -0.001	<0.001
MMed score as categorical (Medium adherence)	Body mass index	-0.086	-0.11, -0.06	<0.001

**Multivariable associations between each mediator and the obesity-related cancer adjusted for the exposure**				
**Fully adjusted model[2]**	**Mediators as continuous**	**HR**	**95% CI**	**P-value**
Cox regression w/continuous mediator	Waist-to-hip ratio	3.02	2.41-3.81	<0.001
Cox regression w/continuous mediator	Body mass index	1.03	1.02-1.03	<0.001

© 2025 Aguilera-Buenosvinos I et al. *JAMA Network Open.*

**Multivariable association between the exposure and the outcome to calculate de control direct effect (CDE) adjusting for each mediator.**					
**Fully adjusted model[2]**	**Exposure[1]**	**Mediators as continuous**	**HR**	**95% CI**	***P-value***
Cox regression adjusted for WHR	MMed score as categorical (Medium adherence)	Waist-to-hip ratio	0.94	0.91-0.98	0.006
Cox regression adjusted for BMI	MMed score as categorical (Medium adherence)	Body mass index	0.94	0.91-0.99	0.01

[1]Score based on the traditional Mediterranean diet score proposed by Trichopoulou et al. (2005). [2]Multivariable adjusted model for the fully multivariable model stratified by country, sex, and age at recruitment (in 1-year categories), and adjusted for attained level of education (primary school, technical school, secondary school, or university degree), physical activity (inactive, moderately inactive, moderately active, and active), smoking status(never smoker, former smoker, and current smoker) and intensity at recruitment , height, alcohol intake (g/day) and total energy intake (Kcal/day) and prevalence of diabetes (yes/no).

© 2025 Aguilera-Buenosvinos I et al. *JAMA Network Open.*

eTable 10. Results From Mediation Analyses for Exploring Mediating Effects Between Adherence to the Mediterranean Diet Score (High vs low adherence) and Obesity-Related Cancers (ORCs) by Baseline Continuous Mediators: BMI and WHR in the EPIC Study

Effect	Causal Estimate	SE	Lower bound	Upper bound	P value
**WHR mediator**					
Rcde	1	2.04E-12	1	1	0.982
Rpnde	1	1.13E-12	1	1	0.976
Rtnde	1	1.34E-12	1	1	0.976
Rpnie	1	1.15E-13	1	1	0.99
Rtnie	1	9.76E-14	1	1	0.968
Rte	1	1.23E-12	1	1	0.976
ERcde	3.21E-12	2.04E-12	-3.61E-12	3.59E-12	0.982
ERintref	-1.44E-12	9.15E-13	-1.60E-12	1.63E-12	0.982
ERintmed	3.28E-13	2.07E-13	-3.75E-13	3.66E-13	0.982
ERpnie	-1.8E-13	1.15E-13	-2.17E-13	2.112E-13	0.99
ERcde(prop)	1.68	0.48	1.06	2.20	0.014
ERintref(prop)	-0.75	0.62	-1.40	-0.05	0.042
ERintmed(prop)	0.17	0.14	0.011	0.30	0.042
ERpnie(prop)	-0.09	0.16	-0.21	0.08	0.126
pm	0.074	0.23	-0.10	0.27	0.114
int	-0.58	0.48	-1.10	-0.04	0.042
pe	-0.68	0.48	-1.20	-0.06	0.044
**BMI mediator**					
Rcde	1	4.62E-12	1	1	0.98
Rpnde	1	1.69E-12	1	1	0.972
Rtnde	1	1.79E-12	1	1	0.972
Rpnie	1	5.30E-14	1	1	0.978
Rtnie	1	4.63E-14	1	1	0.976

© 2025 Aguilera-Buenosvinos I et al. *JAMA Network Open.*

Rte	1	1.74E-12	1	1	0.97
ERcde	7.30E-12	4.62E-12	-8.35E-12	8.21E-12	0.98
ERintref	-4.67E-12	2.93E-12	-5.14E-12	5.22E-12	0.97
ERintmed	1.53E-13	9.67E-14	-1.84E-13	1.68E-13	0.97
ERpnie	-8.66E-14	5.30E-14	-9.81E-14	1.04E-13	0.97
ERcde(prop)	2.70	1.67	1.39	4.16	0.02
ERintref(prop)	-1.73	1.73	-3.19	-0.41	0.03
ERintmed(prop)	0.05	0.06	0.011	0.10	0.03
ERpnie(prop)	-0.03	0.08	-0.07	0.035	0.13
pm	0.02	0.10	-0.04	0.10	0.118
int	-1.67	1.67	-3.10	-0.40	0.038
pe	-1.70	1.67	-3.1	-0.39	0.038

eTable 10 (continued). Results From Mediation Analyses for Exploring Mediating Effects Between Adherence to the Mediterranean Diet Score (High vs low adherence) and Obesity-Related Cancers (ORCs) by Baseline Continuous Mediators: BMI and WHR in the EPIC Study

Effect	Causal Estimate	SE	Lower bound	Upper bound	P value
**WHR mediator**					
Rcde	1	1.9E-12	1	1	0.976
Rpnde	1	1.20E-12	1	1	0.994
Rtnde	1	1.47E-12	1	1	0.99
Rpnie	1	1.74E-13	1	1	0.994
Rtnie	1	1.44E-13	1	1	0.946
Rte	1	1.31E-12	1	1	0.988

© 2025 Aguilera-Buenosvinos I et al. *JAMA Network Open.*

ERcde	2.91E-12	1.99E-12	-3.8E-12	3.73E-12	0.976
ERintref	-1.11E-12	8.10E-13	-1.53E-12	1.52E-12	0.978
ERintmed	3.88E-13	2.79E-13	-5.18E-13	5.42E-13	0.976
ERpnie	-2.8E-13	1.74E-13	-3.08E-13	3.16E-13	0.994
ERcde(prop)	1.52	10.55	0.26	2.92	0.048
ERintref(prop)	-0.58	11.25	-2.52	0.87	0.11
ERintmed(prop)	0.20	3.81	-0.29	0.89	0.11
ERpnie(prop)	-0.14	4.42	-0.78	0.88	0.31
pm	0.055	3.96	-0.82	1.32	0.41
int	-0.37	7.41	-1.71	0.57	0.112
pe	-0.52	10.5	-1,92	0,73	0,118
**BMI mediator**					
Rcde	1	4.10E-12	1	1	0.994
Rpnde	1	1.56E-12	1	1	0.996
Rtnde	1	1.75E-12	1	1	0.982
Rpnie	1	1.22E-13	1	1	0.99
Rtnie	1	9.92E-14	1	1	0.962
Rte	1	1.64E-12	1	1	0.996
ERcde	5.47E-12	4.10E-12	-7.7E-12	8.04E-12	0.994
ERintref	-3.37E-12	2.55E-12	-4.97E-12	4.80E-12	0.98
ERintmed	2.52E-13	1.93E-13	-3.53E-13	3.78E-13	0.978
ERpnie	-1.99E-13	1.22E-13	-2.25E-13	2.21E-13	0.99
ERcde(prop)	2.54	10.81	-0.31	6.5	0.062
ERintref(prop)	-1.56	11.3	-5.69	1.60	0.104
ERintmed(prop)	0.117	0.82	-0.12	0.42	0.104

© 2025 Aguilera-Buenosvinos I et al. *JAMA Network Open.*

ERpnie(prop)	-0.09	1.3	-0.59	0.37	0.346
pm	0.02	1.43	-0.46	0.54	0.372
int	-1.44	10.51	-5.22	1.48	0.104
pe	-1.54	10.81	-5.50	1.31	0.112

Abbreviations :
cde (controlled direct effect), pnde (pure natural direct effect), tnde (total natural direct effect), pnie (pure natural indirect effect), tnie (total natural indirect effect), te (total effect), intref (reference interaction), intmed (mediated interaction), cde(prop) (proportion cde), intref(prop) (proportion intref), intmed(prop) (proportion intmed), pnie(prop) (proportion pnie), pm (proportion mediated), int (proportion attributable to interaction) and pe (proportion eliminated).Rcde (controlled direct effect ratio), Rpnde (pure natural direct effect ratio), Rtnde (total natural direct effect ratio), Rpnie (pure natural indirect effect ratio), Rtnie (total natural indirect effect ratio), Rte (total effect ratio). ERcde (excess ratio due to controlled direct effect), ERintref (excess ratio due to reference interaction), ERintmed (excess ratio due to mediated interaction), ERpnie (excess ratio due to pure natural indirect effect).

ERcde(prop) (proportion excess ratio due to controlled direct effect), ERintref(prop) (proportion excess ratio due to reference interaction), ERintmed(prop) (proportion excess ratio due to mediated interaction), ERpnie(prop) (proportion excess ratio due to pure natural indirect effect).

© 2025 Aguilera-Buenosvinos I et al. *JAMA Network Open.*

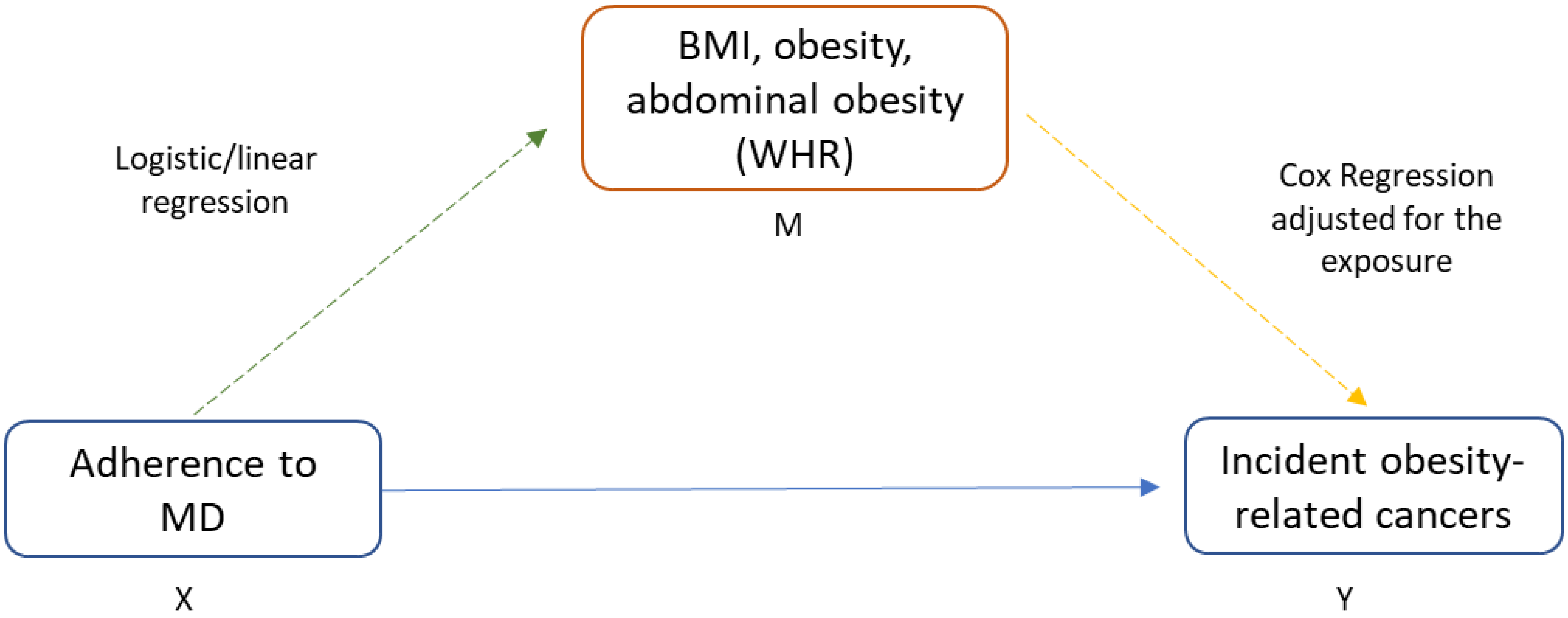

eFigure. DAGs for Exploring Whether the Association Between Mediterranean diet score and ORC Could Be Partially Mediated by Body Mass Index Within the EPIC Cohort Study

© 2025 Aguilera-Buenosvinos I et al. *JAMA Network Open.*
