# Supplementary material for: Adherence to the Mediterranean Diet and Obesity-Linked Cancer Risk in EPIC
Source: JAMA Netw Open. 2025 Feb 25;8(2):e2461031. doi: 10.1001/jamanetworkopen.2024.61031 (PMC11862969; doi:10.1001/jamanetworkopen.2024.61031)
Supplement: Supplement 2. — Data Sharing Statement [file jamanetwopen-e2461031-s002.pdf]

Data Sharing Statement

Aguilera-Buenosvinos. Adherence to the Mediterranean Diet and Obesity-Linked Cancer Risk in EPIC. *JAMA Netw Open*. Published February 20, 2025.
doi:10.1001/jamanetworkopen.2024.61031

Data
Data available: No

Additional Information
Explanation for why data not available: The data and results derived from the analysis of this proposal are based on the European EPIC cohort whose database is managed by the IARC institution. Permission is granted by IARC. The main author of the article was able to make use of these data during her doctoral stay. It is a database that is not freely accessible.
